# Early results of low-level laser application for masticatory muscle pain: a double-blind randomized clinical study

**DOI:** 10.1186/s12903-015-0116-5

**Published:** 2015-10-23

**Authors:** Erkan Sancakli, Bilge Gökçen-Röhlıg, Ali Balık, Değer Öngül, Selin Kıpırdı, Haluk Keskın

**Affiliations:** Department of Prosthodontics, Faculty of Dentistry, Istanbul University, Capa, 34390 Istanbul, Turkey

**Keywords:** Masticatory muscle pain, Low-level laser, Direct irradiation

## Abstract

**Background:**

To evaluate the effect of Low Level Laser (LLL) application at the points of greatest pain in patients with chronic masticatory muscle pain.

**Methods:**

A total number of 30 (21 women, 9 men, with a mean age of 39.2) were selected after the diagnosis of MPDS according to the Research Diagnostic Criteria for Temporomandibular Disorder (RDC/TMD). The patients were randomly divided into three groups; laser group I (*n* = 10); patients received the LLL at the point of greatest pain, laser group II (*n* = 10); patients received LLL at pre-established points in the effected muscles and placebo group (*n* = 10). LLL and placebo were applied three times per week, for a total of 12 sessions. Mandibular mobility was examined, masticator muscles tenderness were assessed and PPT values were obtained. Subjective pain levels were evaluated using VAS. The measurements performed before the treatment and after the completion of the therapy. Descriptive statistics (mean, standard deviation, and frequency) Student’s *t*-test, Mann–Whitney *U*-test and paired-sample *t*-tests were used for analysis.

**Results:**

In both laser groups, there was a statically significant reduction in PPT values of the muscles, number of muscles without any pain on palpation increased significantly, mandibular movements’ ranges were improved. Laser group I demonstrated statistically better results than the Laser group II in all of the measured values. Plasebo group did not show any statistically difference in any of the measured values.

**Conclusions:**

LLLT can be accepted as an alternative treatment modality in the management of masticatory muscle pain and direct irradiation seems to effect better.

**Trial registration:**

Current Controlled Trials ISRCTN31085, Date of registration 28/08/20145.

## Background

Temporomandibular disorders (TMDs) are probably the most common cause of pain of non-dental origin in the maxillofacial area [1]. The category of TMDs embraces a number of clinical problems related to masticator muscles, temporomandibular joint (TMJ) and associated structures, or both [2]. Such disorders are characterised by pain, joint sounds, and restricted mandibular movement. The incidence of TMD ranges from 40 to 75 % in general populations [3] and approximately 65 % of affected patients experience associated pain [4]. This great number of patients is treated using different modalities, depending on the aetiology of signs and symptoms. Conservative, rather than aggressive and irreversible, treatment is preferred to relieve symptoms, diminish pain, and re-establish function [5, 6]. As the aetiology of TMD is multifactorial, available treatments are extensive and diverse, including the use of occlusal splints, low-level laser therapy (LLLT), and transcutaneous electrical nerve stimulation, among many others. Given that better therapeutic results (i.e. pain relief) are obtained with the combined use of modalities, several recent studies have examined the use of LLLT to reduce TMD pain and promote biostimulation effects [7–9].

LLLT, also known as ‘soft laser therapy’, has been investigated and used in the treatment of musculoskeletal pain syndromes for more than 3 decades. Numerous studies have demonstrated that LLLT effectively relieves pain and helps to re-establish normal function in patients with TMD [7–10]. Interest in this method is probably attributable to its ease of use and low cost, although it is time consuming. LLLT has been found to have analgesic, myorelaxant, tissue healing, and biostimulation effects [7–9]. The mechanisms of LLLT essentially rely on particular visible red and infrared light waves in photoreceptors within sub-cellular components, particularly the respiratory chain within mitochondrial membranes [11, 12]. This light absorption causes activation of the respiratory chain and oxidation of the nicotinamide adenine dinucleotide hydrogen pool, which leads to a complex metabolic process culminating in the stimulation of normal cell function of the cell.

The body of literature on LLLT is large, but the results of randomised and non-randomised clinical studies (including those with double-blind designs) are controversial. The protocols used in clinical studies have varied in terms of power intensity and location of laser application. Although a 632.8-nm neon-helium laser [13]. and 660-nm [14], 780-nm [14, 15], 810-nm [16], 904-nm [17], and 830–904-nm [10] soft lasers have been used and reported to be effective, the most common wavelength in therapeutic use is probably 810–830 nm [18–21]. No consensus has been reached on the choice of laser application sites for the treatment of muscle pain. Some studies have employed laser application only at pre-established points in the affected muscles [13, 22] or acupuncture points [23], whereas few studies [10, 24, 25] have targeted the point of greatest pain. Evidence for the best laser application site for the treatment of TMD of muscular origin is insufficient. Thus, the aim of this study was to investigate the efficacy of LLLT application at different sites in the treatment of TMD of muscular origin. We expected that direct application of LLLT at the point of greatest pain would reduce muscle pain to a greater degree than would application at pre-established points and placebo.

## Methods

This study was performed at the Department of Prosthodontics, School of Dentistry, University of Istanbul, Turkey. Patients with orofacial pain who reported to the school’s primary TMD referral centre were selected over a period of approximately 13 months (September 2010 - October 2011). Sample selection was based on standardised clinical examination using the following inclusion criteria: 1) diagnosis of myofascial pain according to the Research Diagnostic Criteria for Temporomandibular Disorder (RDC/TMD) [26], 2) age 18–60 years, and 3) natural posterior occlusion. Exclusion criteria were: 1) disc displacement with reduction or without reduction with or without limited opening, arthralgia, arthritis, or arthrosis; 2) general inflammatory connective tissue disease (e.g. rheumatoid arthritis); 3) psychiatric disorder; 4) tumour; 5) hearth disease or pacemaker; 7) pregnancy; 8) symptoms that could be referred to other orofacial region diseases (e.g. toothache, neuralgia, migraine); 9) treatment or medication use for headache or bruxism in the last 2 years; and 10) local skin infection over the masseter muscle. The Ethics Committee of the Istanbul University Faculty of Medicine approved the study protocol. All participants received a full explanation of the study and provided written informed consent.

### Study protocol

This study was a double-blind randomised clinical trial (RCT). Participants were divided randomly into 3 age- and sex-matched groups: laser group I (LGI), laser group II (LGII) and the placebo group (PG). The randomizations of the patients were done with the help of a computer program. Patients were unaware of their group assignments. All participants underwent three treatment sessions per week for 1 month (total, 12 sessions). An experienced prosthodontist who was blinded to the applied treatment evaluated the patients twice: 30 min before the first laser application session and 30 min after the last laser application session. Evaluation consisted of 1) functional examination, 2) pressure pain threshold (PPT) measurement, and 3) subjective pain intensity measurement.

One week before the study began and during the course of the study, patients were asked not to take any analgesic or receive pain treatment. At the end of the study, patients in the PG received suitable treatment, such as an occlusal appliance or physical therapy.

#### Laser exposure

A continuous low-intensity semiconductor (Doris Diode Laser, CTL 1106 MX; Warsaw, Poland) was used for laser exposure. This device generates continuous radiation with regulated power. The single-probe laser device applies a laser diode generating infrared radiation of 820 nm wavelength. The beam diameter of the device is 6 mm and the probe has an angle of 45°. The energy intensity given to each muscle point was adjusted to 3 J/cm^2^ by applying 300 mW output power for 10 s.

In all groups, the laser was applied at a 2-mm distance. In LGI, LLLT was applied precisely and continuously to the greatest points of pain in the related muscle (masseter and/or temporalis). In LGII, LLLT was applied in the same manner to three predetermined points on the masseter muscle (superior [MS], middle [MM], and inferior [MI] points) and three points on the temporalis muscle (anterior [TA], middle, and posterior points). In the PG, the laser device was switched on, but not programmed.

#### Functional examination

Functional examination was based on Kurkcu’s official Turkish translation of the RDC/TMD [26]. The same prosthodontist examined all patients after calibration using the RDC/TMD as the gold standard.

Masticatory muscle tenderness was assessed bilaterally by palpation. Mobility of the mandible on opening, lateral excursions, and protrusion was measured with a plastic millimetre-scale ruler. Alterations in the opening pathway were also evaluated. Patients were asked to report any pain during muscle palpation and mandibular movements, and this information was recorded using a verbal scale: 0, no pain; 1, mild pain; 2, moderate pain; and 3, severe pain.

#### Pressure pain threshold measurement

After calibration, an experienced prosthodontist used a dial algometer (Pain Test™ Model FPK; Wagner Instruments, Greenwich, CT, USA) to measure PPTs (kg/cm^2^) on the masticatory muscles. Compressions were performed using a 1-cm^2^ rubber tip. After performing a few test measurements on each participant’s lower arm, PPT values were obtained before and after treatment by applying pressure to the bilateral MS, MM, MI, and TA points. Patients were instructed to state immediately when the feeling of pressure became painful, at which time the pressure was stopped. Measurements were separated by 30-s rest periods.

#### Pain intensity measurement

The participants were asked to evaluate their overall pain. Their subjective pain levels (mm) were evaluated using a 100-mm visual analogue scale (VAS) before and after treatment.

### Statistical analysis

Under the assumption of a difference of one standard deviation in the primary endpoint among groups, an alpha level of 5 %, and a power goal of 80 %, 10 patients per group were determined to be necessary. For all statistical tests, NCSS 2007 and PASS 2008 Statistical & Power Analysis Software (NCSS, Kaysville, Utah, USA) were used. Descriptive statistics (mean, standard deviation, and frequency) were calculated for all variables. Quantitative data were compared using Student’s *t*-test for normally distributed parameters and the Mann–Whitney *U*-test for non-normally distributed parameters. Within-group comparison of normally distributed parameters was performed using paired-sample *t*-tests. The results were analysed using 95 % confidence intervals, with a significance level of *p* < 0.05.

## Results

Sixty-two of 814 examined patients with TMD of muscular origin fulfilled the inclusion criteria, and 33 of these patients agreed to participate in the study. Three enrolled patients did not attend appointments regularly and were excluded from the study. The study sample thus comprised 30 patients with TMD of muscular origin (21 women, 9 men; mean age, 39.2 ± 2.8 years) allocated to the three study groups (*n* = 10 per group). We identified no significant difference in age, sex, or pain duration among groups. Participants’ demographic characteristics are shown in Table 1.

**Table 1:** Demographic data of the groups

		LGI	LGII	PG	*p*
		Mean ± SD	Mean ± SD	Mean ± SD	
Age		30,80 ± 9,81	29,33 ± 8,59	31,94 ± 12,20	^*a*^ *0.531*
Gender	Male	3	3	3	^*b*^ *0.373*
	Female	7	7	7	
Pain duration (months)		10.5 ± 1.7	9.8 ± 1.1	10.2 ± 1.9	^*a*^ *0.289*

^*a*^ *Oneway ANOVA Test*

^*b*^ *chi-square test*

Participants in all groups reported the most severe pain in the masseter muscle. Laser exposure significantly reduced pain on palpation and (Table 2). Pain severity also decreased slightly in the PG (Table 2). It demonstrates the percentage of the pain intensity of the examined muscles before and after real laser exposure and placebo laser application in each group. The intensity of pain was reduced more in LGI than in LGII. PPTs of the examined muscles increased significantly in the LLLT groups, but not in the PG (Table 3). Although the difference between LGI and LGII was not statistically significant, LGI demonstrated slightly better results. Vertical movement, lateral excursions, and protrusion also improved significantly in the LLLT groups (Table 4). Pre- and post-treatment VAS scores differed significantly in the LLLT groups, but not in the PG (Table 5). No patient reported any adverse effect related to laser application during or after the treatment period.

**Table 2:** The results of muscle palpations

	LGI		LGII		LGII	
	*n* (%)		*n* (%)		*n* (%)	
TA	Before treatment	40	Before treatment	40	Before treatment	50
	After treatment	30	After treatment	30	After treatment	50
TM	Before treatment	40	Before treatment	40	Before treatment	40
	After treatment	30	After treatment	20	After treatment	30
TP	Before treatment	10	Before treatment	40	Before treatment	20
	After treatment	10	After treatment	40	After treatment	20
MS	Before treatment	80	Before treatment	80	Before treatment	70
	After treatment	40	After treatment	60	After treatment	70
MM	Before treatment	90	Before treatment	80	Before treatment	90
	After treatment	60	After treatment	50	After treatment	80
MI	Before treatment	80	Before treatment	90	Before treatment	80
	After treatment	40	After treatment	50	After treatment	70

*TA* temporalis anterior, *TM* temporalis middle, *TP* temporalis posterior, *MS* masseter superior, *MM* masseter middle, *MI* masseter inferior

**Table 3:** Comparison of PPT (kg/cm^2^) values. Each line demonstrates the statistical analysis of a muscle before and after laser exposure

	Laser Group I			Laser Group II			Placebo Group		
	M (kg/cm^2^)	SD	*p*	M (kg/cm^2^)	SD	*p*	M (kg/cm^2^)	SD	*p*
*TA*	30.55	4.98	0.017532*	31.55	4.82	0.027532*	34.32	5.67	0.27757 **
*TM*	33.42	5.37	0.00922 *	35.42	5.71	0.02022 *	36.97	4.36	0.52936 **
*TP*	32.12	5.71	0.00574 *	36.50	5.67	0.00274 *	38.07	4.46	0.26742 **
*MS*	22.75	5.52	0.00756 *	23.11	5.86	0.00956 *	25.50	6.00	0.55225 **
*MM*	20.87	4.87	0.00203 *	19.45	4.44	0.00303 *	23.60	4.72	0.94166 **
*MI*	23.22	5.11	0.00032 *	19.22	5.05	0.00062 *	26.10	5.52	0.70732 **
*SCM*	21.77	5.80	0.00031 *	21.50	4.48	0.00061 *	24.75	5.95	0.95113 **

Student *t* test was used

*M* mean, *SD* Standard deviation, 95 % confidence interval, * *p* < 0.05 statistically significant, ** *p* ≥ .05 statistically non-significant

*TA* temporalis anterior, *TM* temporalis middle, *TP* temporalis posterior, *MS* masseter superior, *MM* masseter middle, *MI* masseter inferior, *SCM* sternocleidomasteideous

**Table 4:** Mandibular movements. Each line demonstrates the statistical analysis of a mandibular movement after the assigned treatment and placebo application

	Laser Group I			Laser Group II			Plasebo Group		
	M (mm)	SD	*p*	M (mm)	SD	*p*	M (mm)	SD	*p*
UOP	38.3	4.59	0.00317*	37.9	4.78	0.00217*	40.1	3.87	0.34084**
MUO	39.9	2.94	0.00865*	38.1	3.00	0.00925*	38.7	2.64	0.26096**
MAO	40.4	5.09	0.00259*	38.0	5.23	0.00822*	39.3	4.89	0.26551**
LLE	5.9	1.20	0.00171*	4.9	0.20	0.00051*	5.7	1.14	0.06001**
RLE	6.05	1.50	0.00065*	5.11	1.70	0.00365*	6.08	1.60	0.08266**
P	5.35	1.38	0.00036*	5.01	1.68	0.00136*	5.22	1.58	0.53079**

Student *t* test was used

*M* mean, *SD* Standard deviation, 95 % confidence interval, * *p* < 0 .05 statistically significant, ** *p* ≥ 0.05 statistically non-significant)

*UOP* unassisted opening without opening, *MUO* maximum unassisted opening, *MAO* maximum assisted opening, *LLE* left lateral excursion, *RLE* right lateral excursion, *P* protrusion

**Table 5:** VAS scores according to groups

	Before Treatment	After Treatment
	Mean ± SD	Mean ± SD
Laser Group I	62.65 ± 10.42	31.46 ± 7.14*
Laser Group II	58.38 ± 7.25	44.05 ± 7.14*
Plasebo Group	53.31 ± 8.79	49.75 ± 9.54**

*Mann–Whitney for differences before /after

*p < 0.05, statistically significant

**p ≥ 0.05 statistically non-significant

## Discussion

The present study demonstrated that the tested type of LLLT, applied bilaterally, was beneficial in the treatment of TMD of muscular origin. Patients who received LLLT showed significant improvements in the tested parameters, whereas those in the PG did not. However, the study hypothesis that direct application of a low-level laser to the sites of most severe pain would produce better results than laser application at predetermined points was not supported. Direct application appeared to be slightly superior to traditional point irradiation, but differences were not significant.

Several previous clinical studies have investigated the effects of LLLT on TMD of myogenic origin using objective and subjective measures of PPTs, mandibular movements, and muscle pain on palpation [7–9]. The documented variation in these effects can be explained by many factors, including methodological differences among studies (e.g. use of placebo, double-blind design, sample characteristics) and LLLT parameters (e.g. application point, wavelength, power output, energy intensity, exposure duration). Furthermore, the effect of a low-level laser increases with the depth of penetration into musculoskeletal tissues. Wavelength and energy density play important roles in light penetration and absorption. In the current study, a light with 820 nm wavelength, 3 J/cm^2^, and 300 mW output was used because these parameters have been reported to be effective for muscle disorders [10]. Gallium-arsenide lasers have been reported to penetrate to depths of 1–5 cm in soft tissue [27], which should be adequate to achieve a therapeutic effect in muscle disorders. The absence in the present study of a significant benefit of direct laser application to the points of greatest muscle pain, which we hypothesised would more effectively target affected tissue, may be due to the limited size of the treated muscle, which resulted in considerable similarity of such targeting to the treatment of predetermined points. In a muscle with larger area and greater thickness, such targeted application might achieve better results.

Pain was the chief complaint of patients in the current study. Pain levels were evaluated subjectively (with a VAS) at the beginning and end of the study. In clinical trials designed to investigate the efficacy of therapies aiming to resolve or reduce pain, the primary endpoint is reduction in pain intensity [28]. For chronic pain, most measures of treatment response involve patient-reported outcomes, as patients are the most important judges of whether changes are relevant [29–31]. The evaluation of pain is difficult because it is not controlled in clinical research; this complex procedure requires the assessment of biological, structural, functional, and emotional pain experiences. Recent chronic pain studies have recommended the evaluation of pain using subjectively reported primary measures [28, 29]. However, clinical assessment should also involve an objective and quantitative evaluation of pain, such as PPT measurement, which enhances the quality of data and enables outcome standardisation and comparison. In the present study, objective functional measures based on the RDC/TMD and PPT values were recorded with the aid of an algometer to accompany patient-reported pain intensity data. PPT values supported the VAS results.

In 2004, the World Association of Laser Therapy approved an agreement on the design of clinical studies employing LLLT; the use of a PG was established as a mandatory component [http://waltza.co.za/wpcontent/uploads/2012/08/walt_standard_for_conduct_of_randomized_controlled_trials.pdf]. Accordingly, an age- and sex-matched PG was included in the present study. The PG showed slight, but not significant, improvement in all tested parameters. These improvements could be explained by many factors. The most likely explanation involves the positive reactions of patients to technologically advanced laser treatment. Furthermore, palpation, which was a treatment component for all study subjects (including those in the PG), can relieve pain [32].

The present study does have some limitations. The present study examined the effect of specific dose of LLLT on pain release and mandibular function and found out that active laser is superior to sham application. However the only the short-term effects were investigated. Additional research should be conducted in order to investigate the long-term effects of LLL in the treatment of TMD. Furthermore only one type of laser settings was used. Different light intensities might have produced different or better results. In order to be able to suggest a treatment protocol, a study population with greater sample size, followed-up for longer period and treated with different low-level laser parameter should be used.

## Conclusions

Within the limitations of the present clinical trial, the tested type of LLLT (820 nm, 3 J/cm^2^, 300 mW output power) showed positive effects in managing pain caused by TMD and improving mandibular function due to its analgesic and myorelaxant effects. The localisation of laser application (at predetermined points *vs*. points of greatest pain), however, did not affect the results of LLLT. Further double-blind, randomised, placebo-controlled clinical research is needed to establish whether these findings have clinical relevance.
